# Integration of molecular cytogenetics, dated molecular phylogeny, and model-based predictions to understand the extreme chromosome reorganization in the Neotropical genus *Tonatia* (Chiroptera: Phyllostomidae)

**DOI:** 10.1186/s12862-015-0494-y

**Published:** 2015-10-06

**Authors:** Cibele G. Sotero-Caio, Marianne Volleth, Federico G. Hoffmann, LuAnn Scott, Holly A. Wichman, Fengtang Yang, Robert J. Baker

**Affiliations:** Department of Biological Sciences, Texas Tech University, Lubbock, TX USA; Department of Human Genetics, Otto-von-Guericke University, Magdeburg, Germany; Department of Biochemistry, Molecular Biology, Entomology, and Plant Pathology, Mississippi State University, Mississippi, MS USA; Institute for Genomics, Biocomputing and Biotechnology, Mississippi State University, Mississippi State, MS USA; Department of Biological Sciences, University of Idaho, Moscow, ID USA; The Wellcome Trust Sanger Institute, Wellcome Trust Genome Campus, Cambridge, UK

**Keywords:** Chromosome evolution, Chromosomal mutation, Karyotypic megaevolution, Phyllostomidae, Centromere, Transposable element

## Abstract

**Background:**

Defining factors that contributed to the fixation of a high number of underdominant chromosomal rearrangements is a complex task because not only molecular mechanisms must be considered, but also the uniqueness of natural history attributes of each taxon. Ideally, detailed investigation of the chromosome architecture of an organism and related groups, placed within a phylogenetic context, is required. We used multiple approaches to investigate the dynamics of chromosomal evolution in lineages of bats with considerable karyotypic variation, focusing on the different facets contributing to fixation of the exceptional chromosomal changes in *Tonatia saurophila*. Integration of empirical data with proposed models of chromosome evolution was performed to understand the probable conditions for *Tonatia*’s karyotypic evolution.

**Results:**

The trajectory of reorganization of chromosome blocks since the common ancestor of Glossophaginae and Phyllostominae subfamilies suggests that multiple tandem fusions, as well as disruption and fusions of conserved phyllostomid chromosomes were major drivers of karyotypic reshuffling in *Tonatia*. Considerable variation in the rates of chromosomal evolution between phyllostomid lineages was observed. Thirty–nine unique fusions and fission events reached fixation in *Tonatia* over a short period of time, followed by ~12 million years of chromosomal stasis. Physical mapping of repetitive DNA revealed an unusual accumulation of LINE-1 sequences on centromeric regions, probably associated with the chromosomal dynamics of this genus.

**Conclusions:**

Multiple rearrangements have reached fixation in a wave-like fashion in phyllostomid bats. Different biological features of *Tonatia* support distinct models of rearrangement fixation, and it is unlikely that the fixations were a result of solely stochastic processes in small ancient populations. Increased recombination rates were probably facilitated by expansion of repetitive DNA, reinforced by aspects of taxon reproduction and ecology.

**Electronic supplementary material:**

The online version of this article (doi:10.1186/s12862-015-0494-y) contains supplementary material, which is available to authorized users.

## Background

How chromosomal rearrangements become established in natural populations has been a recurrent question in evolutionary cytogenetics for decades [1–3]. The absence of “the one model” of chromosomal fixation can be attributed to the amount of variables associated with cellular, molecular, ecological, and population distinctiveness of each taxonomic group where chromosome rearrangements have become established [4]. Because individual models usually focus on specific aspects of chromosomal change, each containing its own set of variables and assumptions, none is widely accountable for all patterns observed in nature. The widespread model predicts that chromosome rearrangements resulting in underdominance require small population sizes to achieve fixation [1, 5]. However, some observed patterns are difficult to reconcile with this single model, such as cases of karyotypic megaevolution, where there is a major re-patterning of the chromosomal segments due to a large number of multiple types of chromosomal rearrangements [6]. In order to understand karyotypic evolution in a given taxon, it is necessary to contrast the observed data with all available models, and then define what combination of circumstances most likely explains the observations. It is noteworthy that some models of chromosomal speciation intrinsically bear assumptions applicable to models of rearrangement fixation within a population (see [7] for a review).

Because of their high levels of karyotypic variation, phyllostomid bats are an ideal group to investigate the mechanisms responsible for chromosome evolution and stasis. This group arose in the Eocene, and has diversified into 11 distinct subfamilies, with over 57 genera and 200 species [8, 9]. The varying rates of karyotypic change predicted for morphologically divergent lineages, coupled with their ample set of biological differences, depict a scenario in which highly rearranged karyotypes might have been established by different means in species with distinct life histories. The subfamily Phyllostominae is an example of karyotypic variation among closely related species, with diploid numbers (2*n*) ranging from 16 to 34 [10]. Most of the species within this group present a karyotype of 2*n* = 32; however, the two species in the genus *Tonatia* (*T. bidens* and *T. saurophila*) have a 2*n* = 16 karyotype that is heavily rearranged relative to other members of the subfamily, including the closely related genus, *Lophostoma* [11, 12]. Because no homology could be detected, based on G-band comparison, to the karyotypes of closely related genera, *Tonatia* was proposed as an example of a megaevolved karyotype [6].

Molecular cytogenetic techniques, such as cross-species chromosome painting, allow the detection of chromosome homologies based on conservation at DNA sequence level, and thus are robust tools to estimate the magnitude of chromosomal change, especially in groups for which classical G-banding comparison have failed to detect interspecific chromosomal homologies. Moreover, by integrating chromosome painting data, molecular dating, and an explicit phylogenetic framework, one can infer evolutionary trajectories and estimate rates of fixation of chromosome rearrangements within particular lineages [13]. The resulting information, together with the mapping of other DNA sequences on the karyotype of closely related taxa, is valuable to test the predictions of different proposed models for fixation of chromosome rearrangements and provides a starting point to unravel the underlying forces shaping karyotypes of extant species.

The goal of this study was to investigate the radical reshuffling of the *Tonatia* karyotype. To do so, we 1) used cross-species chromosome painting to map the chromosomal homologies between *T. saurophila* and six phyllostomid species from four subfamilies (Macrotinae, Phyllostominae, Glossophaginae, and Lonchophyllinae); 2) reconstructed phylogenetic relationships using molecular data to define ancestral and derived syntenic associations on the karyotypes of these subfamilies; and, 3) used molecular time estimates to provide a temporal framework for the observed chromosomal changes. Our data allowed us to estimate the minimum number of rearrangements required to derive the extant karyotype of *T. saurophila* from the proposed ancestral Phyllostominae condition, as well as the number of unique fusion and fission events in each lineage since the divergence of two distinct subfamilies (Glossophaginae/Phyllostominae). In addition, probes of repetitive sequences were mapped onto the genomes of Phyllostominae bats to investigate their role as potential drivers of chromosome reshuffling within the group. The probability of fixation of *Tonatia* chromosome rearrangements is discussed in light of different models, integrating data on fluorescence *in situ* hybridization (FISH), long-term effective population sizes (N_e_), selection coefficients for specific rearrangements, and natural history aspects of the genus.

## Methods

### Chromosome preparations and G-banding

Chromosome preparations were obtained from bone marrow or tissue culture following the methods described by Baker et al. [14], and in accordance with animal welfare guidelines established by the Texas Tech University Animal Care and Use Committee. Voucher specimens, tissues, and cell suspensions are deposited at the Natural Science Research Laboratory (NSRL) of the Museum of Texas Tech University, under the following identification numbers: *Tonatia saurophila* (TSA) from Honduras, Ecuador, and Costa Rica: TK101463♀, TK104519♂, TK104616♀, TK104655♀, TK167809♀; *Lophostoma occidentalis* (LOC) from Ecuador: TK104520♂ and TK104505♀; *Mimon crenulatum* (MCR) from Ecuador: TK104437♀, TK104615♀, TK104516♂, TK104620♀, TK135711♂, TK135714♀, TK135715♀. G-banding technique was carried out following Seabright [15], with trypsin (0.25 %), incubation times varying from 15 to 30 min at 37 °C.

### Chromosome painting

Whole chromosome paint probes from *Macrotus californicus* (MCA), generated by DOP-PCR of flow-sorted chromosomes [16], were used for the chromosome painting experiments on metaphase chromosomes of *T. saurophila, L. occidentalis, and M. crenulatum. In situ* hybridizations were carried out according to Yang et al. [17] and Volleth et al. [18]. Cy3-conjugated streptavidin (Amersham Biosciences, 1:1000) was used for detection after 72 h of hybridization. The chromosomes were counterstained with DAPI (4’,6-diamidino-2-phenylindole). To distinguish MCA chromosomes 8, 10, and 13, we have used *Myotis myotis* chromosome 12 specific paint, which corresponds to MCA 8. No hybridization with MCA Y chromosome paint was performed. Chromosome painting results for Glossophaginae and Lonchophyllinae species from Sotero-Caio et al. [16] were integrated in the analysis.

### FISH with repetitive DNA

Our repetitive DNA FISH (fluorescence *in situ* hybridizations) included the use of 45S ribosomal DNA (rDNA), long interspersed element 1 (LINE-1) and telomeric (TTAGGG)_n_, sequences as probes. The rDNA sequences were amplified from genomic DNA of *Noctilio albiventris*, using the following18S and 28S specific primers: 28S F-1: 5’ GCC GAA ACG ATC TCA ACC TAT T 3’; 28S R-1: 5’ GAG CCA ATC CTT ATC CCG AA 3’; 18S F-1: 5’TCA ACT TTC GAT GGT AGT 3’; and 18S R-1: 5’GCA AGC TTA TGA CCC GCA CTT A 3’. The LINE-1 probe used (Tbid8b) was isolated from *T. saurophila* genome by degenerate PCR amplification of a 575 bp portion of ORF2 straddling the reverse transcriptase domain, followed by cloning and enrichment for LINE-1 fragments retaining a single open reading frame, i.e., the youngest elements in the *T. saurophila* genome [19, 20]. The rDNA PCR products and the isolated LINE-1 clones were labeled with biotin using the Bionick^™^ DNA Labeling System (Molecular Probes®) or with digoxigenin using the DIG-Nick Translation Mix (Roche). Hybridizations were performed following the FISH protocol described by Raudsepp and Chowdhary [21]. Commercially available ready-to-use human chromosome Pan-telomeric probes (Star*FISH©, Cambio) were used according to manufacturer’s instructions.

### Phylogenetic analyses

We performed Bayesian and Maximum Likelihood (ML) phylogenetic analyses using available sequences from the mitochondrial *cytochrome-b* and *12S*–*16S* rRNA fragment, as well as a portion of the nuclear *RAG2* gene, using the Hoffmann et al. [22] dataset, adding representatives of *Glossophaga* to the ingroup, and representatives of *Noctilio* and *Pteronotus*, as outgroup sequences. Due to missing data for *L. occidentalis*, four other species of *Lophostoma* were used to estimate the relationships between this genus and other phyllostomine (Additional file 1: Table S1) Sequences for each fragment were aligned using MUSCLE [23], and concatenated prior to phylogenetic estimation. Bayesian estimation was done in MrBayes version 3.1.2 [24], running four simultaneous chains for 5 × 10^7^ generations, sampling trees every 2,500 generations, and using default priors. Convergence was accessed by measuring the standard deviation of the split frequency among parallel chains. Chains were considered converged once the average split frequency was lower than 0.01. We recovered a majority-rule consensus of the last 2,500 trees collected after convergence was reached, and discarded trees collected before convergence. We implemented a 7-partition analysis where each codon position in the nuclear *RAG2*, the mitochondrial *cytochrome-b*, and the rDNA fragment had an independent GTR + Γ model of nucleotide substitution. The best-fitting models of nucleotide substitution for partition were independently selected using the corrected Akaike Information Criterion [25, 26]. Maximum-likelihood searches were done in Treefinder version June 2007 [27], following similar methods used in Bayesian analyses. Node support was estimated with 1,000 bootstrap replicates [28].

### Divergence dating

Molecular time estimation was performed in BEAST v2.1.3 [29] according to Khan et al. [30] to estimate divergences within *Noctilio*. A lognormal molecular clock with an independent model of nucleotide substitution for each separate partition was implemented. Because the node corresponding to the Mormoopidae/Phyllostomidae split is dated to the late Oligocene, it was constrained to have a minimum age of 28.3 million years (myr) with an exponential mean of 3.2, allowing the maximum age of this divergence to occur ~40.6 myr ago [31, 32]. Node dates were estimated using a birth–death process prior as proposed for reconstructing phylogenies without fossil lineages [33]. Analyses consisted of multiple independent runs for a total of 150 × 10^6^ iterations, with every 1,000th iteration logged for all analyses. Independent runs were combined for each data set using LogCombiner version 2.1.3 [34]. TRACER version 1.6 [35] was used to determine appropriate burn-in (10 %), and examine convergence, effective sample sizes (ESSs), and 95 % highest probability density intervals (HPD) of constrained priors. Due to missing data, a shorter alignment from the *RAG2* dataset was used to estimate divergence time since the split of the two *Anoura* from the Glossophaginae common ancestor.

### Determination of ancestral karyotypes

We integrated the chromosome painting data of the present work and Sotero-Caio et al. [16] to determine the syntenic associations present in the karyotype of the Phyllostominae and Glossophaginae common ancestor (PGA), as well as to determine syntenic associations or chromosomal blocks that were present at the basal node of each subfamily. Using *M. californicus* as outgroup, syntenic associations present in species from the two subfamilies were parsimoniously placed in the PGA karyotype. Chromosome morphology and banding patterns were evaluated to uncover synapomorphic inversions.

### Rates of chromosomal evolution

The rates of fixation of unique chromosome rearrangements for Phyllostominae and Glossophaginae species were calculated using the estimated number of fixed unique chromosomal changes divided by the amount of time required to derive the karyotypes in each particular lineage. The magnitude of fixed unique chromosomal changes per species was obtained using two major parameters: 1) number of unique fusions, calculated as the number of fusion events from the ancestral subfamilial state required to form unique syntenic associations in the species analyzed; and, 2) number of required fissions to derive the number of chromosome blocks on extant karyotypes, calculated using the syntenic associations of *M. californicus* and the number of blocks on the proposed ancestral subfamilial karyotype as the outgroups. We decided not to include other karyotypic change parameters in this analysis, such as inversions or centromeric shifts, due to the subjectivity of banding pattern comparison resulting from the differential G-banding quality of different preparations. The time component was calculated using internodal divergence times obtained from BEAST analysis. Standard errors for the time estimates were calculated from the upper and lower confidence intervals of each node, allowing the inference of the upper and lower rates of fixation of chromosome rearrangements.

### Models of fixation of chromosome rearrangements and long-term effective population size estimations

The model of fixation of chromosome rearrangements from Lande [5] was used to estimate average long-term effective population size (N_e_) from chromosomal data for TSA and also *Anoura cultrata* (ACU) for comparisons. We have used three selection coefficient values (s = 0.1, 0.3, and 0.5), for differential selective pressures/disadvantage on heterozygous conditions to account for a combination of different types of rearrangements presented by these species. A spontaneous chromosomal mutation rate (u) range of 10^−3^ to 10^−4^ was selected to accommodate for the variation proposed for mammals ([5] and references therein). Rates of fixation of chromosomal change (R) to be incorporated in this method were estimated using the size-corrected average generation length data estimated by Pacifici et al. [36] to calculate R in terms of fixation of rearrangements per species per generation. Since estimations of divergence times from molecular data are broad, we included upper and lower values of our date estimates to account for the obtained standard errors and find corresponding N_e_ variations. We contrasted the obtained results with the predictions of Chesser and Baker [37], which test the feasibility of Lande’s model, taking bat biological characteristics into consideration. Additional theoretical models were examined in light of the current knowledge of TSA biological features.

## Results

### Hybridizations and chromosome characterization of *Tonatia saurophila* (TSA)

**Fig. 1:**
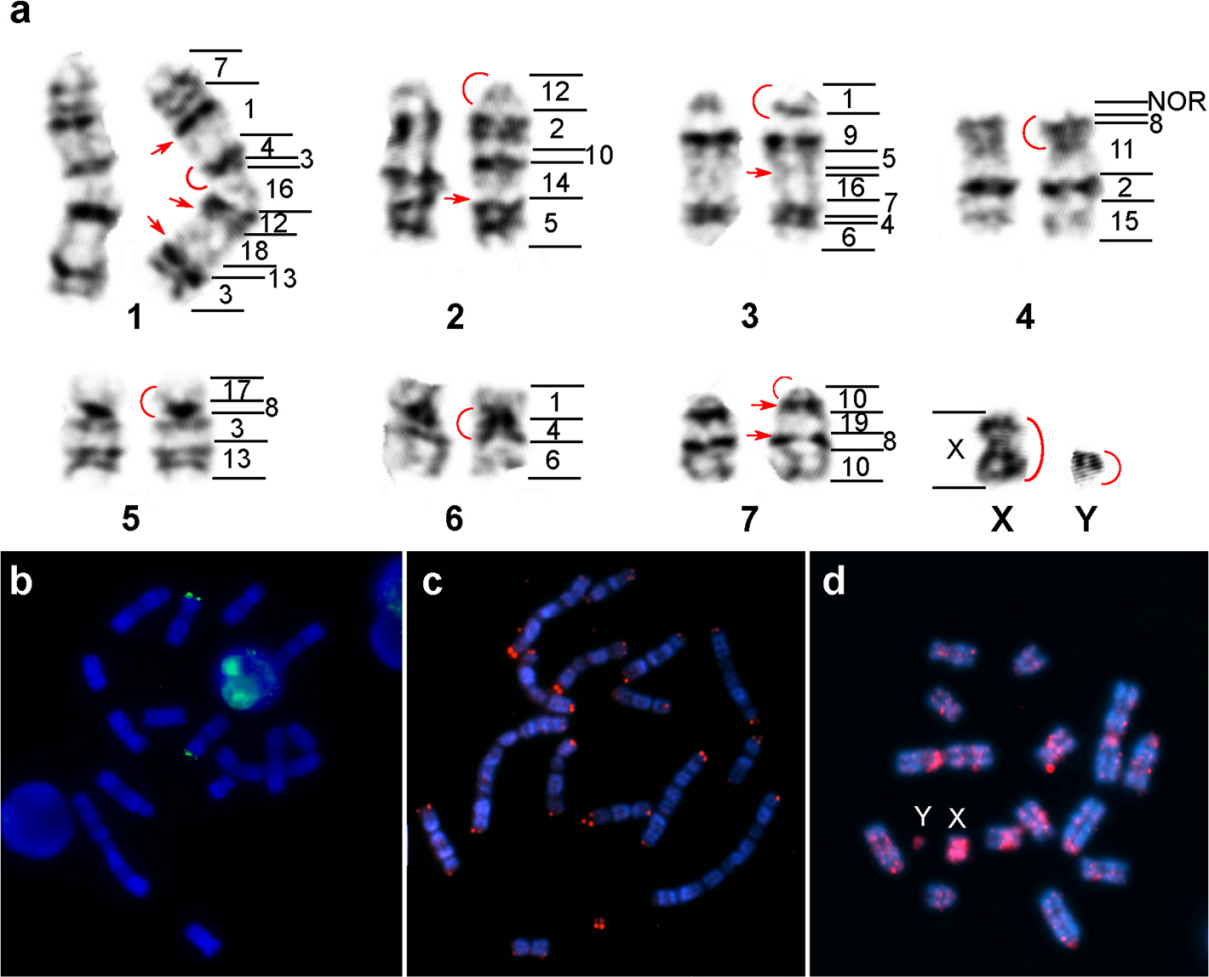
In situ hybridizations on *Tonatia saurophila* (TSA) karyotype. Schematic representation of MCA chromosome homologies on the G-banded karyotype of TSA (**a**). MCA corresponding chromosome number is shown to the right of each autosome pair. Mitotic metaphases showing rDNA (**b**), telomeric (**c**), and portion of LINE-1 (Tbid8b) sequences location on TSA chromosomes (**d**). Red arrows and arcs in (**a**) delimit the regions of LINE-1-sequence amplification

The karyotype of *Tonatia saurophila* from Ecuador and Costa Rica had diploid and fundamental numbers of 2n = 16, FN = 22, respectively (Fig. 1). This chromosomal formula differs from the reported karyotype of 2n = 16, FN = 20 of specimens from Trinidad and Brazil by a pericentric inversion on the fourth largest autosome [10, 12, 38]. The hybridizations with MCA whole chromosome paints detected a total of 36 pairs of homologous segments on the autosomes of TSA. With the exception of the allosomes, no chromosome of MCA was conserved as an individual chromosome in the TSA genome. Additionally, the syntenies of most MCA chromosomes appeared disrupted on the TSA karyotype, except for seven chromosome pairs, which seemed to have fused to other chromosomes as entire syntenic blocks (MCA 9, 11, 14, 15, 17, 18, and 19). The assignment of MCA homologous segments on the G-banded karyotype of TSA is shown in Fig. 1a.

The hybridizations with repetitive DNA probes (Fig. 1b–d) revealed different patterns: 1) the major cluster of 45S rDNA is located at the distal portion of the short arm of TSA chromosome 4; 2) telomeric DNA was detected distally on all chromosome arms; and, 3) the probe of young *Tonatia* LINE-1 sequences hybridized to the centromeres of all TSA chromosomes, to the negative G-band regions of some autosomes, apparently corresponding to breakpoint regions between different MCA syntenic blocks, as well as to the entire length of the sex chromosomes. Additionally, we found LINE-1 signal in a region on TSA 3, between MCA 5 and 16, whereby no MCA or PHA [12] homology have been detected.

### Hybridizations and chromosome characterization of *Lophostoma occidentalis* (LOC)

**Fig. 2:**
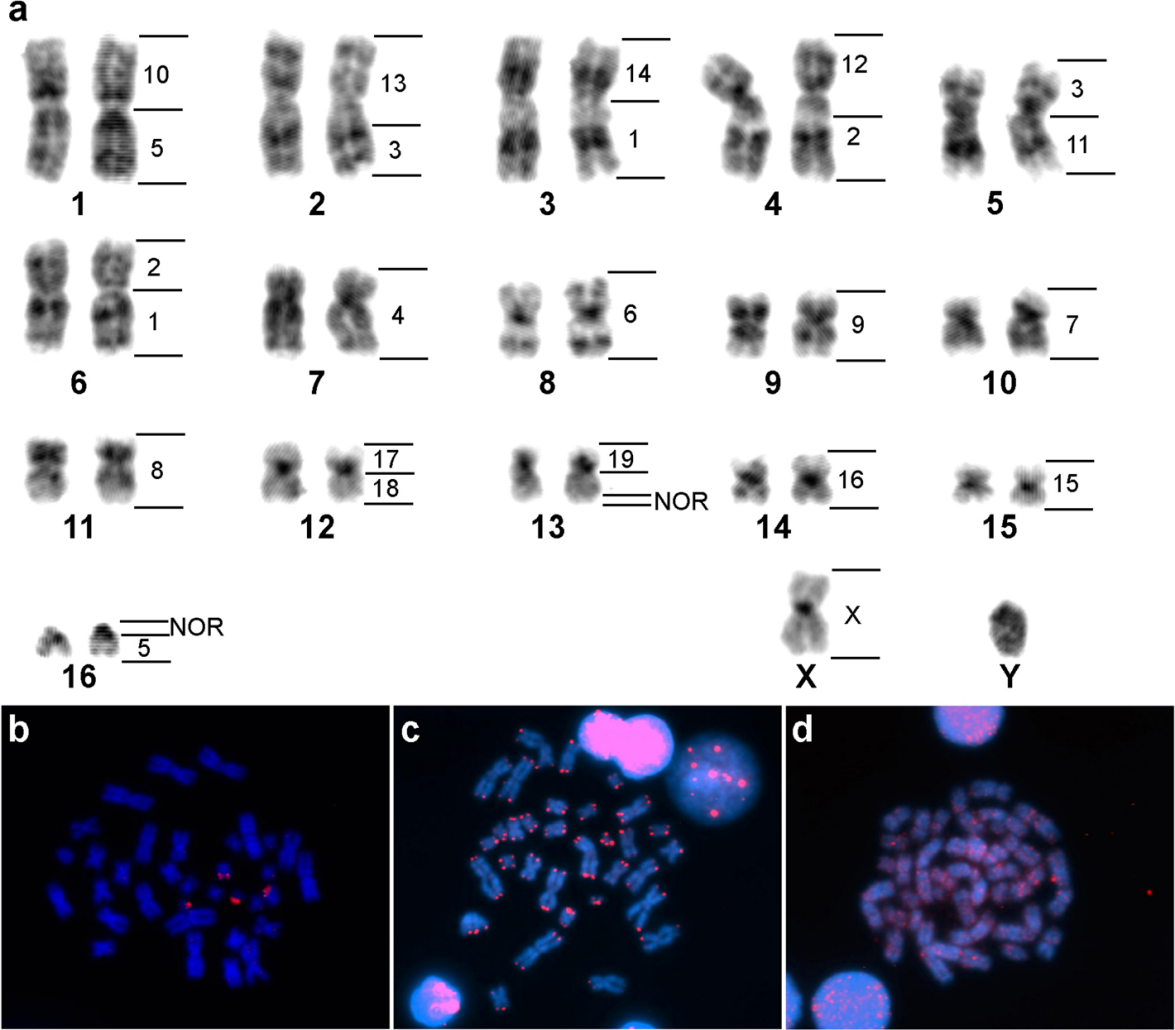
In situ hybridization results on *Lophostoma occidentalis* (LOC) chromosomes. G-banded karyotype of LOC showing a summary of MCA chromosomal homologies to the right of each chromosome pair (**a**). FISH showing the location of rDNA (**b**), telomeric (**c**), and Tbid8b LINE-1 (**d**) sequences on LOC mitotic chromosomes

The karyotype of *Lophostoma occidentalis* is comprised of 17 chromosome pairs (2n = 34) with FN = 62, as described in Baker et al., [39] see Velazco and Cadenillas [40] for taxonomic status and species diagnosis. This karyotype is identical to the one reported for the species *L. silvicolum* [12]. A total of 23 pairs of homologous segments were detected by FISH with the MCA autosome probes (Fig. 2a). The rDNA FISH has revealed nucleolar organizing regions (NORs) on two LOC chromosome pairs: on the distal portion of LOC 13q and LOC 16p. Telomeric sequences were located at all chromosome termini, whereas hybridizations with *Tonatia* LINE-1 have not revealed any differential accumulation of these sequences in any particular chromosome or chromosomal region (Fig. 2b–d).

### Hybridizations and chromosome characterization of *Mimon crenulatum* (MCR)

**Fig. 3:**
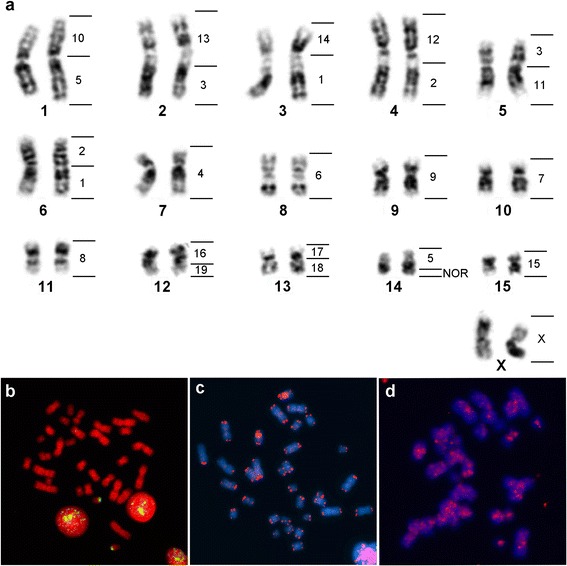
In situ hybridization results on *Mimon crenulatum* (MCR) karyotype. Inverted DAPI-banded karyotype of MCR showing identified regions of homology to MCA chromosomes (**a**). Hybridization sites and MCA chromosome numbers are presented to the right of each chromosome pair. FISH signals on mitotic metaphases of MCR with rDNA (**b**), telomeric (**c**), Tbid8b LINE-1 (**d**) probes

The specimens of *Mimon crenulatum* analyzed herein had 2n = 32, with either FN = 59 or 60. The individual with a FN = 59 was a heterozygote for two chromosomal inversions, encompassing one of the chromosomes of pair five (acrocentric vs. submetacentric) and one of pair 6 (subtelocentric vs. submetacentric) (Fig. 3). The individuals with FN = 60 had a submetacentric morphology for both MCR 5 and 6 (data not shown). A single pair of NORs was detected distally on the long arm of MCR 14 (Fig. 3b) whereas the telomeric FISH detected the telomeres of all chromosomes, plus one non-telomeric site on the centromeric region of MCR 7 (Fig. 3c). Hybridizations with the young LINE Tbid8b did not reveal consistent accumulation patterns for these sequences (Fig. 3d). Representative images of the chromosome painting on the three species are presented on (Additional file 2: Figure S1).

### Phylogenetic relationships and divergence dates of Phyllostominae

The relationships of the studied taxa revealed by the Bayesian analysis are similar to those reported by Hoffmann et al. [22]. Within Phyllostominae, the nodes for tribes Phyllostomini, Macrophyllini and Vampyrini (sensu Baker et al. [41]) were recovered. Within Phyllostomini, *Tonatia* diverged first, followed by the radiation of lineages that gave rise to four genera, including *Mimon* and *Lophostoma*. The tree topology with the estimated divergence dates and lineage name abbreviations are presented in the (Additional file 3: Figure S2).

### Phyllostominae, Glossophaginae, and PGA ancestral karyotypes

**Fig. 4:**
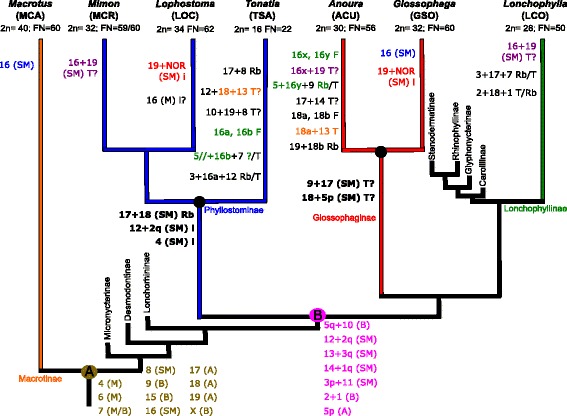
Ancestral and convergent chromosome associations mapped on the Phyllostomidae tree using the *Macrotus californicus* chromosome numbering system. Based on the recurrence of the same syntenies among the species analyzed, we were able to infer the syntenic associations present as individual chromosomes at the base of the common ancestor of *Macrotus* and the remaining phyllostomids (node A), as well as the common ancestor of Phyllostominae, Glossophaginae, and Lonchophyllinae (PGA, node B). The chromosomes present at A have remained conserved at B. Synapomorphies for Glossophaginae and the synapomorphies of Phyllostominae are mapped in black bold font at their respective nodes. The proposed convergent syntenic associations of chromosomes MCA16 – 19 are depicted in the same color for different species on terminal branches of the tree. The abbreviations beside the derived chromosomal states indicate proposed rearrangements required to generate them: T (telomeric fusion), i (inversion), Rb (Robertsonian translocation), F (fission). Multiple types of rearrangements required to the formation of specific chromosomal associations are separated by a (/), and question marks (?) represent rearrangements that were not able to be defined. The abbreviations in parenthesis correspond to the chromosomal morphology as follows: A (acrocentric) B (biarmed), M (metacentric), SM (submetacentric). Letters beside a chromosome segment represent the ancestral short (p) or long (q) chromosome arm or are used to identify fission segments with uncertain arm origin (a, b, x, and y)

Several conserved chromosomes and chromosomal blocks were identified between the analyzed species (Fig. 4, Additional file 4: Table S2). Seven chromosomes (including a submetacentric X chromosome) were shared by all phyllostomids analyzed and were present at the most basal family node, as well as the PGA karyotype. The syntenic blocks corresponding to MCA 16 through 19 were prone to homoplasy, but were able to define their ancestral states using MCA as the outgroup. The different states of MCA 16, 17, 18, and 19 on the karyotypes of the analyzed species are presented in a phylogenetic context in Fig. 4. Seven additional syntenic segments were synapomorphic for Phyllostominae, Glossophaginae and Lonchophyllinae. The above information allows an estimation of a PGA karyotype comprised of 36 chromosomes.

The comparative analysis among the Phyllostominae (TSA, LOC, and MCR) has shown considerable karyotypic conservation between LOC and MCR. Their karyotypes share most of the syntenic blocks identified by chromosome painting, except for the differential linkage of MCA 16 and 19. Interestingly, the karyotype of *Glossophaga soricina* (GSO, Glossophaginae) also presents most of the syntenies retained by LOC and MCR. Because of the observed conservatism, we propose that the Phyllostominae and Glossophaginae ancestral karyotypes were essentially those of LOC and GSO, respectively (synapomorphies for each subfamily are shown in Fig. 4). Three Phyllostominae synapomorphies, inversion (i) of MCA 4, 12 + 2qi, and 17 + 18) were confirmed by G-band comparison with the karyotype of *Trachops cirrhosus* (Phyllostominae) as a Phyllostomini outgroup [42].

### Rates of chromosomal evolution and long-term effective population size estimations: chromosome evolution of TSA (Phyllostominae) and ACU (Glossophaginae)

TSA and ACU had the highest values of unique fusions and fissions (39 and 14, respectively) required to derive their karyotypes from their respective ancestral subfamilial karyotypes. We estimate that at least 13 autapomorphic tandem fusions have contributed to the reorganization of TSA karyotype. The magnitude of unique fixed changes for MCR consisted of a single fission event, not including the two polymorphic inversions, whereas no changes from the ancestral subfamilial chromosome complement were observed for GSO and LOC (Table 1, Additional file 4: Tables S3–S5). The rates of fixation of chromosome rearrangements varied for the different branches across the phylogenetic tree, with periods of chromosomal stasis versus evolution (Table 1). The standard errors for the ages of distinct branches ranged from 1.2 to 3.4 myr and contributed to wide variations in R (fixation events over time) values, especially for lineages where chromosome stasis occurred. The fixation of a large number of chromosome rearrangements in the lineage that gave rise to the genus *Tonatia* occurred in a relatively short period of time (6 ± 2.7 myr), followed by chromosome stasis for the remaining 12 ± 1.7 myr after divergence of the two species within the genus. Similarly, a short period of time of 4.6 ± 3.2 myr was estimated for the fixation of 14 unique changes in the lineage that gave rise to the genus *Anoura* from the glossophagine ancestor. The two cases described above differ from the general trend observed for most of the branches analyzed, in which less than two fixation events occurred for periods of time spanning up to 12 myr.

**Table 1:** Differential rates of fixation (R) of unique fusions and syntenic group disruptions (fissions) during the evolution of the analyzed lineages

Lineage	Time (myr)	SE	Fission/Fusions		R (changes/myr)		Comments
				Lower		Upper	
PGA to PA	1.7006	3.4345	1	−0.5756	0.5880	0.1947	Stasis
PA to PiA	3.9046	3.1613	0	0	0	0	Stasis
PiA to L/M	0.6135	2.9292	0	0	0	0	Stasis
L/M to M	3.0248	2.8037	1	4.5228	0.3306	0.1715	variable
L/M to L	10.1032	1.2038	0	0	0	0	Stasis
PiA to T	6.2324	2.7562	39	11.22	6.26	4.34	Evolution
T to TSA/TBI	12.6332	1.7908	0 – 1	0 – 0.0922	0 – 0.0791	0-0.0693	Stasis
PGA to GA	7.786	3.3742	2	0.4533	0.2568	0.1792	Stasis
GA to A^a^	4.6205	3.2150	14	9.9609	3.03	1.7867	Evolution
A to ACA/AGE^a^	6.7802	2.0390	0	0	0	0	Stasis

The time used corresponds to specific internodal distances in million years (myr) and the lower and upper values of R were calculated using the standard errors for the divergence time of each branch (SE). The two species of *Tonatia* as well as all *Anoura* species have the same karyotype

*Abbreviations* as follows: Phyllostominae + Glossophaginae ancestor (PGA), Phyllostominae ancestor (PA), Glossophaginae ancestor (GA); Phyllostomini ancestor (PiA); *Lophostoma* + *Mimon* ancestor (L/M); genus *Lophostoma* (L), genus *Mimon* (M); genus *Tonatia* (T); genus *Anoura* (A); *Tonatia bidens* (TBI); *Tonatia saurophila* (TSA); *Anoura caudifer* (ACA); *Anoura geoffroyii* (AGE). The date estimates of the branches marked with an ^a^ were derived from *RAG2* sequence data only.

**Fig. 5:**
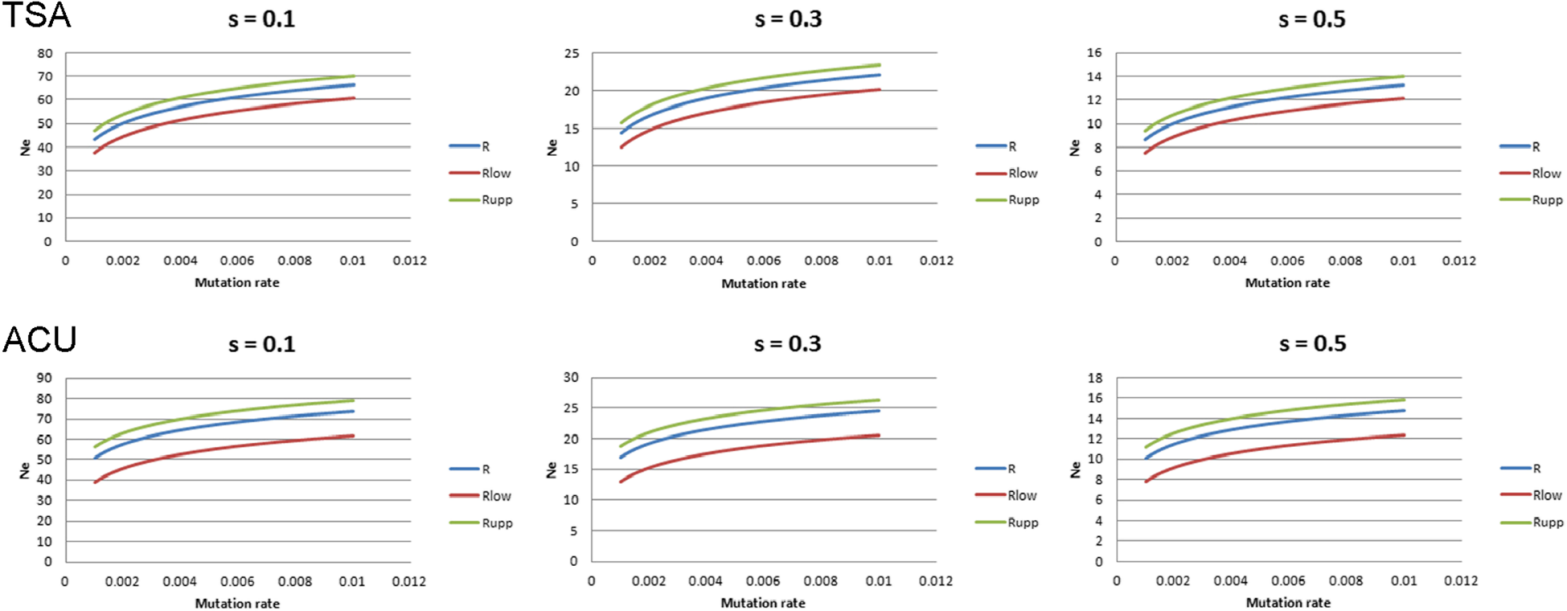
Results of long-term effective population size (N_e_) analysis. The graphs show the N_e_ of TSA (top) and ACU (bottom) as a function of mutation rate, calculated from the rates of fixation of chromosomal rearrangements for each species using Lande’s 1979 model. Upper and lower curves correspond to the N_e_ values obtained with adjusted rates using the standard errors calculated for the divergence dates shown in Table 1. s-values correspond to the selection pressure of rearrangements in heterozygotes

The results of the N_e_ analyses for TSA and ACU are summarized in Fig. 5, and the detailed calculations are presented in the Additional file 5: Table S6. As expected from Lande’s model [5], long-term effective population sizes required for the fixation of the number of rearrangements presented by the two species are low (less than 70 and 80 individuals for TSA and ACU, respectively), even when the lower and upper values of fixation times are considered. For selection coefficients greater than 0.3, N_e_ values are significantly reduced to lower than 24 for TSA and 27 for ACU, considering the highest rates of mutation for the range used.

## Discussion

### Extensive and conservative chromosomal evolution in Phyllostominae and Glossophaginae

Our results show that the rates of chromosomal evolution since the divergence of the lineages that gave rise to the analyzed species were not constant over time, and that the set of rearrangements leading to the chromosomal constitution of genera such as *Tonatia* (Phyllostominae) and *Anoura* (Glossophaginae), have occurred as the result of waves of rearrangements specific to those lineages. On the other hand, little chromosomal evolution was observed for the lineages leading to the genera *Mimon* and *Lophostoma* (Phyllostominae), and *Glossophaga* (Glossophaginae), since these groups have retained most of the syntenic blocks that were present in the PGA karyotype. G-banding and FISH analyses suggest that the karyotype of other phyllostomine species have not changed considerably from the ancestral Phyllostominae condition proposed herein [6, 12, 43, 44], and that the ancestral Phyllostomidae karyotype is not very different from that presented by the phyllostomine *Phyllostomus hastatus* in terms of syntenic associations [12, 45], implying an overall conservative trend in terms of chromosomal rearrangements within the subfamily. Similarly, *Glossophaga* is nested within a clade comprised of four other genera (*Erophylla*, *Brachyphylla*, *Monophyllus*, and *Leptonycteris*), all of which with identical karyotypes [46]. The chromosomal variation within Glossophaginae seems to be restricted to a monophyletic clade comprising *Anoura* (older origin) and four other relatively recent genera: *Hylonycteris*, *Choeroniscus*, *Choeronycteris*, and *Musonycteris* [6, 8, 47]. These observations suggest that the processes responsible for the extensive chromosome reshuffling in *Tonatia* and *Anoura* might be the result of ephemeral molecular processes, occurring independently in a temporally punctuated fashion.

The unusual high rates of gross chromosomal change of *Tonatia* are comparable to the extreme rates of chromosomal evolution within gibbon genera (22 rearrangements/5myr in *Hoolock*) and can be placed among one of the highest among mammals [48, 49]. For *Tonatia*, a total of 39 unique fission and fusion events have been estimated in the present analysis, however the definition of specific rearrangements as well as determination of the order of fixation events for this lineage proved to be a difficult task. Thus, the number of rearrangements of *Tonatia* and *Anoura* calculated herein might be an overestimation if translocations were an important component of their karyotypic mutation framework (each accounting for a simultaneous fission + fusion). Nevertheless, we estimated that 13 tandem fusions have occurred to derive the karyotype of *Tonatia* from the Phyllostominae ancestor, which would still result in at least 26 rearrangements in that lineage if the remainder rearrangements were all translocation-derived. In addition, refined molecular cytogenetic techniques, such as BAC-FISH, microarrays, and selective sequencing, have shown that results from cross-species chromosome painting might underestimate the total number of rearrangements identified for a given species [48, 50]. Thus, since our analysis does not account for inversions, centromeric shifts, and smaller chromosome translocations, we conclude that the *Tonatia* lineage had one of the highest rates of chromosomal evolution among mammals.

An intriguing aspect of *Tonatia* karyotypic evolution is that regardless of the mechanism responsible for generating these high rates of change, it seems to have ceased after the establishment of the 2n = 16 karyotype and before speciation of the genus. That raises the question of how long natural populations can cope with high rates of change without extinction due to the negative effects on meiosis and before selection drives more intensive control of the underlying mechanisms. Perhaps, an even more relevant question would be how a large number of rearrangements like that observed for *Tonatia* could have achieved fixation within a relatively short period of time. Other bats of the family Phyllostomidae have also undergone multiple rearrangements, resulting in karyotypes with little identifiable G-band arm homologies [6]. These include several non-related lineages from the subfamilies Carolliinae, Glossophaginae, and Stenodermatinae. Family-wide cross-species chromosome painting studies will help unravel lineage-specific rates of rearrangements and their implications for speciation and diversification among phyllostomids. Questions as to why closely related groups can present differential levels of rearrangements will then be able to be addressed.

Finally, we observed that some syntenic blocks are prone to convergence within phyllostomid bats. Examples are the multiple independent fusion/fission events, resulting in slightly different associations in MCR and LOC (Phyllostominae) versus LCO (Lonchophyllinae) and ACU (Glossophaginae). Conversely, the convergent states of MCA 18 might be derived from intense chromosome reorganization on ACU and TSA and less likely are an intrinsic feature of this chromosome.

### Repetitive DNA and evolution of genome architecture in *Tonatia* and other phyllostomids

Repetitive DNA (and duplicated gene families) has been hypothesized to be one of the major drivers of chromosomal change due to their potential to promote non-homologous chromosome exchange through illegitimate recombination between homologous sequences [51–55]. Our chromosome painting and rates of chromosomal change analyses indicated that the patterns of rapid reshuffling of TSA karyotype might be the outcome of non-allelic recombination of such sequences. To better understand the contribution of repetitive sequences in the genomic architecture of TSA, we analyzed the patterns of distribution and accumulation of three classes of repetitive DNA: telomeric, rDNA, and young LINE-1 sequences.

Interstitial telomeric sites (ITS) have been regarded as remnants of chromosomal fusion promoted by repetitive subtelomeric elements [56, 57] as well as inversions [58, 59]. Our telomeric FISH revealed a telomere-only pattern, despite the 13 tandem fusions that contributed to the generation of the highly rearranged karyotype of TSA. This suggests that these sequences might either be absent or in low copy numbers at the identified tandem fusion regions. If subtelomeric DNA was responsible for the rapid reshuffling of *Tonatia* genome, the significant number reduction of these and of telomeric sequences after fusion events might be the critical factor resulting in karyotypic stasis for the last 12 myr. Interestingly, MCR presented telomeric sequences enrichment on the centromere of a chromosome pair that is conserved among several phyllostomine species (MCR 7/ LOC 7). This is a submetacentric element, derived from a metacentric ancestral phyllostomid chromosome (MCA 4). ITS amplification in this particular MCR chromosome is most likely correlated with other types of species-specific repetitive DNA, rolling circle replication, or mechanisms that allow tandem amplification of the canonical telomere sequence within satellite DNA, rather than be derived from gross chromosome rearrangements, such as inversions [60]. Alternatively, the relocation of telomeres by at least two inversions of the ancestral metacentric element might have provided raw material for ITS amplification in MCR that was lost in other species of Phyllostominae.

LINE elements are ubiquitous transposable elements (TEs) in mammalian genomes, and our hybridization results have shown a substantial centromeric buildup of a recently transposed sequence corresponding to partial ORF2 of a *Tonatia*-specific LINE-1 [19], which is not shared by the other two phyllostomine bats analyzed. This LINE-1 centromeric enrichment is uncommon among mammals, for which there is a preferential LINE-1 accumulation on positive G-band regions throughout autosomal length, and especially in sex chromosomes. The centromeric regions, however, are usually devoid of these sequences. The only in situ hybridization study of LINE-1 distribution on bat chromosomes has shown that at least for one phyllostomid species, *Carollia brevicauda*, the distribution of LINE-1 sequences follows this widespread pattern [52, 61–65]. The pattern seen in *Tonatia*, however, resembles the LINE-1-Alu-SVA (LAVA) gibbon-specific TE massive centromeric accumulation detailed by Carbone et al. [66]. Carbone et al. [49] have demonstrated for the first time that high rates of gross chromosome rearrangements can be a direct result of premature termination of transcripts of chromosome segregation genes due to insertion of these composite elements.

Accumulation of at least a portion of LINE-1 at TSA centromeric and some breakpoint regions provide a starting point to investigate their potential role as karyotype modifiers in this species. On one hand, it is possible that *Tonatia* LINE-1 sequences have played a role in promoting chromosome translocations through non-homologous recombination. Alternatively, because the probes used correspond solely to a portion of LINE-1, we cannot reject the hypothesis that these sequences are part of composite elements, which could increase levels of rearrangements by affecting expression patterns of chromosome segregation genes [49]. Regardless of their role as karyotypic modifiers, an important aspect of centromeric LINE-1 accumulation observed in TSA chromosomes is their contribution as structural centromeric components and possibly epigenetic regulators of the centromere dynamics in TSA. If part of the centromeric satellite DNA, these sequences are expected to be stable structural components of heterochromatin, serving as binding sites for chromatin remodeling complexes and centromere-related binding factors and having specific amplification dynamics [67]. Centromeric amplification of retroelement-derived repeats has been reported for other mammalian groups with varying degrees of rates of chromosomal evolution, which might display distinct levels of epigenetic repression of TEs [51, 68, 69]. There is no evidence that the probes used in our study differ substantially in structure and composition from sequences isolated from other bats [19, 20] or that phyllostomid bats present an unusual repetitive DNA landscape compared to other mammals [70]. Thus, our results provide an insight into the complexity of TE roles for the chromosomal dynamics within TSA.

Ribosomal genes (rDNA) are unusual repetitive sequences when it comes to patterns of accumulation and dispersal (reviewed by McStay and Grummt [71]). Besides particularities of rDNA evolution, such as concerted evolution and paralog recombination [72], rDNA sites seem to be hotspots for double strand breaks, which are often in physical proximity on non-homologous chromosomes [73]. Because of the abovementioned characteristics, rDNA-bearing chromosomes are proposed to be highly prone to translocations. Our results and the integration of previously published work on FISH with ribosomal DNA and on chromosome painting shows that the distributional pattern of rDNA in phyllostomid bats follows the trends described by Milhomem et al. [74], where lack of association of the major rDNA cluster to specific homologous chromosomes has been reported. We observed the association of NORs to MCA 19 in distantly-related species (LOC and GSO), to MCA 5 in the closely related LOC and MCR, to MCA 8 in TSA, and to MCA 9 in ACU. Similarly, published work using *Phyllostomus hastatus* (PHA) whole chromosome probes have shown rDNA association to different PHA chromosomes, namely PHA 9 and 12 in *Desmodus rotundus*, PHA 15 in PHA and possibly *Diphylla ecaudata* [44, 75, 76], PHA 2 and 13 in *Micronycteris hirsuta* [77], and PHA 5 and X in *Carollia brevicauda* [43, 78]. This trend indicates an independent amplification of rDNA in non-homologous chromosomes in different taxa, and does not support the hypothesis that the rearrangements observed for phyllostomid species were derived from translocations induced by rDNA recombination. Thus, these sequences might not be informative phylogenetic markers on the study of Phyllostomidae evolution.

### Models for the fixation of chromosome rearrangements and their implications for chromosomal evolution in TSA

The integration of our data with a set of published models and the biological features of the studied species allows further investigation of the mechanisms responsible for the fixation of large numbers of chromosomal rearrangements in phyllostomid bats. We first used the classic model of Lande [5] of stochastic fixation of rearrangements to estimate the long-term effective population sizes required to fix the chromosomal rearrangements of TSA and ACU. To our knowledge, this is the first attempt to integrate chromosome painting data and molecular dating to this model. The most important assumption of Lande’s model is the lower fitness conferred to heterozygotes for chromosomal rearrangements, with tandem fusions having a higher associated negative selective pressure than Robertsonian rearrangements [1, 2]. One prediction from this model is that small population sizes would facilitate fixation of chromosomal rearrangements that have a significant negative heterotic effect (drift). Accordingly, TSA and ACU analysis output consisted of small N_e_ values regardless of the selective pressures for the set of rearrangements, underlying chromosomal mutation rates, or estimated fixation rates. The larger values of long-term population sizes, approximately 70 and 80 individuals for TSA and ACU, respectively, would have to be accompanied by large mutation rates and less than 10 % production of unviable gametes by heterozygotes to allow the fixation of the detected rearrangements. The N_e_ values obtained from this model are unrealistic to ensure long-term persistence of a species. Therefore, either rates of chromosome fixation are not appropriate predictors of N_e_, or these species are exceptions with successful population recovery after extended periods of bottlenecks for over 6 myrs. We propose that because our results have shown that the rates of chromosomal rearrangements are not constant over time, they violate one of the major assumptions of Lande’s model (homogeneous rate of fixation of chromosome rearrangements), and thus, this model is a poor predictor of historical population sizes for phyllostomid bats.

According to the model of Chesser and Baker [37], the fixation of chromosomal rearrangements in small populations through drift would only be achieved under the conditions of extremely small population size (less than 10 founders), little underdominance of the rearrangements on heterozygotes, and a high number of offspring per mating. Our results present conflictive situations regarding the two first circumstances because the smaller population size values obtained here are associated with the strongest selection coefficient against the rearrangements (s > 0.5). Thus, if Chesser and Baker’s predictions are accurate, no fixation is possible for TSA for most of our N_e_ values, since population sizes smaller than 20 founders were associated with fecundity reductions greater than 0.25. Additionally, with a single offspring per mating [79], population recovery would not be possible for small demes of less than 10 TSA individuals.

Interestingly, different biological features of *Tonatia* are compatible with different models. For instance, TSA is considered locally rare in terms of abundance, and has only been found in groups of less than 11 individuals with high fidelity to their roosting sites [80, 81], which agrees with predictions of rearrangement fixation through drift in small demes. The potential for daily dispersal of these bats, however, is relatively high, which might indicate that local populations are actually larger due to area coverage [82]. Additionally, some *Tonatia* extant features are compatible to the predictions of the adaptive rearrangement models, in which suppressed recombination might lead to adapted chromosomal forms [83, 84]. These include the omnivorous feeding habit, and current patterns of distribution for the two species which might be widely distributed and adapted to different types of climatic and ecological conditions as a result of phenotypic plasticity provided by ancestral rearrangements.

The simulations of fixation of chromosome rearrangements by Chesser and Baker [37] were performed considering a single initial chromosome rearrangement, and its trajectory of fixation/population size. In the cases of rapid and extensive chromosome reshuffling, new computational models allowing for an input of several concomitant and subsequent rearrangements using fixation rates established with the methods used in this work would be useful to understand for how long polymorphisms for different rearrangements can coexist.

## Conclusions

In summary, it is unlikely that the great number and complexity of TSA chromosomal rearrangements would be fixed solely through drift in natural populations. Thus, we propose alternative scenarios might have played a role to circumvent the negative heterosis in these species. 1) Based on the observations of LINE-1 accumulation for this species, increased recombination [52, 85] among repetitive sequences in this lineage might have led to unprecedented mutation rates. The larger amplification in gene-poor regions, such as centromeres and perhaps telomeres might have led to recurrent breaks involving the same chromosomes in different individuals. This would allow an increased occurrence of homozygote individuals for specific rearrangements, facilitating fixation in large populations. 2) The current adaptive success of *Tonatia* species might be indicative of adaptive karyotypes. We hypothesize that the large number of disrupted euchromatic regions, coupled with suppressed recombination between locally adapted loci in different rearrangement regions would have promoted adaptation and speciation in *Tonatia*. 3) We cannot reject the hypothesis that other factors such as meiotic drive, a phenomenon poorly studied in bats, might have acted synergistically to allow the fixation of *Tonatia* rearrangements. Therefore, we conclude that outcomes of the different models discussed above bring features that act synergistically with ancestral biological features, facilitating the fixation of multiple rearrangements in TSA.

## Additional files

Additional file 1: Table S1. Specimens used in the molecular analysis and respective GenBank accession numbers. (DOCX 24 kb) Additional file 2: Figure S1. Examples of in situ hybridizations using *Macrotus californicus* (MCA) chromosome paints on Phyllostominae metaphases. The MCA chromosome-specific probes used in TSA (a–c), LOC (d–f), and MCR (g–i) metaphases are indicated in pink in the upper-right corner of each picture. (JPEG 315 kb) Additional file 3: Figure S2. Molecular phylogram depicting the relationships among genera of bats in the subfamily Phyllostominae (highlighted in bold) and the divergence time estimates (node ages) from BEAST analysis of all genes. Nodes with posterior probability values higher than 0.98 in (a) are marked with an *. Abbreviations for node names are as follows: Phyllostominae + Glossophaginae ancestor (PGA), Phyllostominae ancestor (PA), Glossophaginae ancestor (GA); Phyllostomini ancestor (PiA); *Lophostoma* + *Mimon* ancestor (L/M); genus *Tonatia* (T); genus *Anoura* (A). (PDF 1529 kb) Additional file 4: Tables S2–S5. Detailed calculations of unique fusions and fissions for ACU and TSA and chromosomal associations shared between the analyzed species. (DOCX 29 kb) Additional file 5: Table S6. Long term effective population size (N_e_) estimations using Lande (1979) model and the calculated rates of chromosomal rearrangement for TSA and ACU. (XLSX 140 kb)

## Abbreviations

2n: Diploid number
ACU: *Anoura cultrata*
DAPI: 4’,6-diamidino-2-phenylindole
DOP-PCR: Degenerate oligonucleotide primed polymerase chain reaction
FISH: Fluorescence *in situ* hybridization
FN: Fundamental number of autosomal arms
GA: Glossophaginae ancestor
GSO: *Glossophaga soricina*
ITS: Interstitial telomeric site
L: genus *Lophostoma*
LAVA: LINE-1-Alu-SVA transposable element
LINE-1: Long interspersed element 1
LCO: *Lonchophylla concava*
LOC: *Lophostoma occidentalis*
L/M: *Lophostoma* + *Mimon* ancestor
M: genus *Mimon*
MCA: *Macrotus californicus*
MCR: *Mimon crenulatum*
ML: Maximum likelihood
Myr: Million years
N_e_: Effective population size
NOR: Nucleolar organizing region
PA: Phyllostominae ancestor
PGA: Phyllostominae + Glossophaginae ancestor
PHA: *Phyllostomus hastatus*
PiA: Phyllostomini ancestor
R: Rate of fixation of chromosome rearrangements
rDNA: ribosomal DNA
s: Selection coefficient
T: genus *Tonatia*
TBI: *Tonatia bidens*
TE: Transposable element
TSA: *Tonatia saurophila*
u: Spontaneous chromosome mutation rate
