# Supplementary material for: Integration of molecular cytogenetics, dated molecular phylogeny, and model-based predictions to understand the extreme chromosome reorganization in the Neotropical genus Tonatia (Chiroptera: Phyllostomidae)
Source: BMC Evol Biol. 2015 Oct 6;15:220. doi: 10.1186/s12862-015-0494-y (PMC4594642; doi:10.1186/s12862-015-0494-y)
Supplement: Additional file 1: Table S1. — Specimens used in the molecular analysis and respective GenBank accession numbers. (DOCX 24 kb) [file 12862_2015_494_MOESM1_ESM.docx]

**Additional file 1 - Table S1: Specimens examined and their locality information.** Specimen number TK49885 is also identified by University of New Mexico voucher number NK30034. Specimens with missing Museum Catalog number have yet to be cataloged. We combined sequences from different individuals within a species, as not all genes were sequenced for the same individuals, and in the cases of *Artibeus* and *Anoura* we combined sequences from closely related species.

| Taxon | Tissue number | Museum catalog number | Country | GenBank accession number | | |
| --- | --- | --- | --- | --- | --- | --- |
|  |  |  |  | *12S–16S rRNA* | *RAG2* | *Cyt-b* |
| *Artibeus concolor* | TK10378 | CMNH63792 | Suriname: Commewijne |  | AF316432 |  |
| Artibeus obscurus | TK17080 | CMNH68951 | Suriname: Nickerie | AY395805 |  |  |
|  | TK104001 | TTU84773 | Ecuador: Pastaza |  |  | DQ869392 |
| *Anoura caudifer* |  | USNM582796 | Peru, Cusco | AY395835 | HG380327* |  |
| Anoura geoffroyi |  | TTU62405 | El Salvador: Santa Ana |  | AF316431 | FJ155495 |
| Chrotopterus auritus | TK17104 | CMNH68638 | Suriname: Saramacca |  | AF316442 |  |
|  | TK21039 | CMNH 76767 | Suriname: Para |  |  | FJ15548 |
|  | TK70457 | MUSM13653 | Peru: Cusco | AF411538 |  |  |
| *Desmodus rotundus* | TK4764 | TTU35582 | México: Guerrero | AF263228 | AF316444 |  |
|  | TK40368 | TTU 61104 | Honduras: Atlantida |  |  | FJ155477 |
| *Diaemus youngi* | TK34625 | TTU62792 | El Salvador: La Paz | AF411534 | AF316445 | FJ155475 |
| *Diphylla ecaudata* | TK13514 | TK13514 | México: Yucatàn | AF411533 | AF316447 |  |
|  | TK13508 | TTU 47509 | Mexico: Yucatan |  |  | FJ155476 |
| *Glossophaga soricina* | TK70461 |  |  | AY395840 |  |  |
|  | TK15311 | TTU33324 | Venezuela: Miranda |  | AF316452 |  |
|  | TK86579 |  | Guyana |  |  | AF382844 |
| *Lonchorhina aurita* | TK20560 | TTU36531 | Mexico, Chiapas | AY395843 | AF316457 | FJ155494 |
| *Lampronycteris brachyotis* | TK25238 | TCWC55445 | Trinidad: Mayaro | AF411536 | AF316463 |  |
|  | TK25239 |  | Trinidad: Mayaro |  |  | AY380748 |
| *Lophostoma brasiliense* | TK18834 | AMNH267103 | French Guyana | AF411544 | AF316489 |  |
|  | TK49898 (F38605) | ROM106608 | Panama |  |  | FJ155486 |
| *Lophostoma evotis* | TK49870 | ROM95626 | México: Campeche | AF411529 |  |  |
|  | TK40341 | TTU61070 | Honduras: Atlantida |  | AF442080 |  |
|  | TK49871 | ROM95625 | Mexico |  |  | FJ155491 |
| *Lophostoma schulzi* | TK18833 | AMNH267106 | French Guyana: Paracou |  | AF442079 |  |
|  | F38318 |  |  |  |  | FJ155485 |
|  | TK49888 | ROM101128 | Locality Missing | AF411532 |  |  |
| *Lophostoma silvicolum* | TK56716 |  | Paraguay: San Pedro | AF442092 | AF442081 | FJ155493 |
| *Lophostoma silvicolum* | TK17946 | CMNH77174 | Suriname: Marowijne | AF263230 |  |  |
|  | TK18832 | AMNH267107 | French Guyana: Paracou |  | AF442083 |  |
|  |  | ROM100949 | Guyana |  |  | FJ155492 |
| *Macrophyllum macrophyllum* | TK19119 | CMNH78289 | Venezuela: Bolivar | AF411540 | AF316458 | FJ155484 |
| *Macrotus waterhousii* | TK32030 | TTU52481 | Cuba: Guantanamo |  | AF316461 |  |
|  | TK32021 | TTU52478 | Cuba: Guantanamo | AF263229 |  |  |
|  | TK27889 | TTU 71435 | Mexico: Morelos |  |  | AY380745 |
| *Micronycteris schmidtorum* | TK70447 | MUSM13737 | Perú: Camisea | AF411535 | AF316470 | AY380753 |
| *Mimon crenulatum* | TK15121 | TTU33287 | Venezuela: Guarico |  | AF316472 |  |
|  | TK25230 | CMNH25230 | Trinidad and Tobago: Trinidad | AF411534 |  | FJ155478 |
| *Noctilio albiventris* | TK86633 |  | Guyana | AF263224 | AF330810 | AF330803 |
| *Noctilio leporinus* | TK10224 |  |  | AF263223 |  | AF330794 |
|  | TK18701 |  |  |  | AF316477 |  |
| *Phylloderma stenops* | TK10201 | CMNH63614 | Suriname: Saramacc | AF411542 | AF316480 |  |
|  | TK86685 |  | Guyana, Berbice District |  |  | FJ155480 |
| *Phyllostomus hastatus* | TK19289 | CMNH19289 | Venezuela: Bolivar | AF411541 |  |  |
|  | TK19243 | CMNH78333 | Venezuela: Bolivar |  | AF316479 | FJ155479 |
| *Pteronotus davyi* | TK25127 | TTU43938 | Trinidad | AF407176 | AF316482 | AF338671 |
| *Tonatia bidens* | TK56633 |  | Paraguay: Dpto. San Pedro | AF442090 | AF442087 | FJ155489 |
| *Tonatia bidens* | TK56519 | MVZ185673 | Brazil: Sao Paulo | AF442091 | AF442088 | FJ155490 |
| *Tonatia saurophila* | TK49889 | ROM103210 | Guyana: Upper Takutu-Upper Essequiba |  | AF442084 | FJ155488 |
|  | TK49892 | ROM104459 | Ecuador: Napo | AF411531 |  |  |
| *Tonatia saurophila* | TK46028 | USNM | Perú: Quebrado |  | AF442085 |  |
|  | TK49890 | ROM103401 | Guyana: Upper Demerara-Berbice | AF411530 |  |  |
|  | TK49895 | ROM104218 | Panama: Canal Zone |  |  | FJ155487 |
| *Trachops cirrhosus* | TK18829 | AMNH267129 | French Guyana: Paracou | AF411539 | AF316490 |  |
|  | TK19132 |  | Venezuela: Bolivar |  |  | FJ155483 |
| *Vampyrum spectrum* | TK40370 | TTU61070 | Honduras: Atlántida: Lancitilla | AF411537 | AF316495 | FJ155482 |

* fragment included in the divergence estimates based on the RAG2 gene only.
