# Supplementary figures and images for: Integration of molecular cytogenetics, dated molecular phylogeny, and model-based predictions to understand the extreme chromosome reorganization in the Neotropical genus Tonatia (Chiroptera: Phyllostomidae)
Source: BMC Evol Biol. 2015 Oct 6;15:220. doi: 10.1186/s12862-015-0494-y (PMC4594642; doi:10.1186/s12862-015-0494-y)

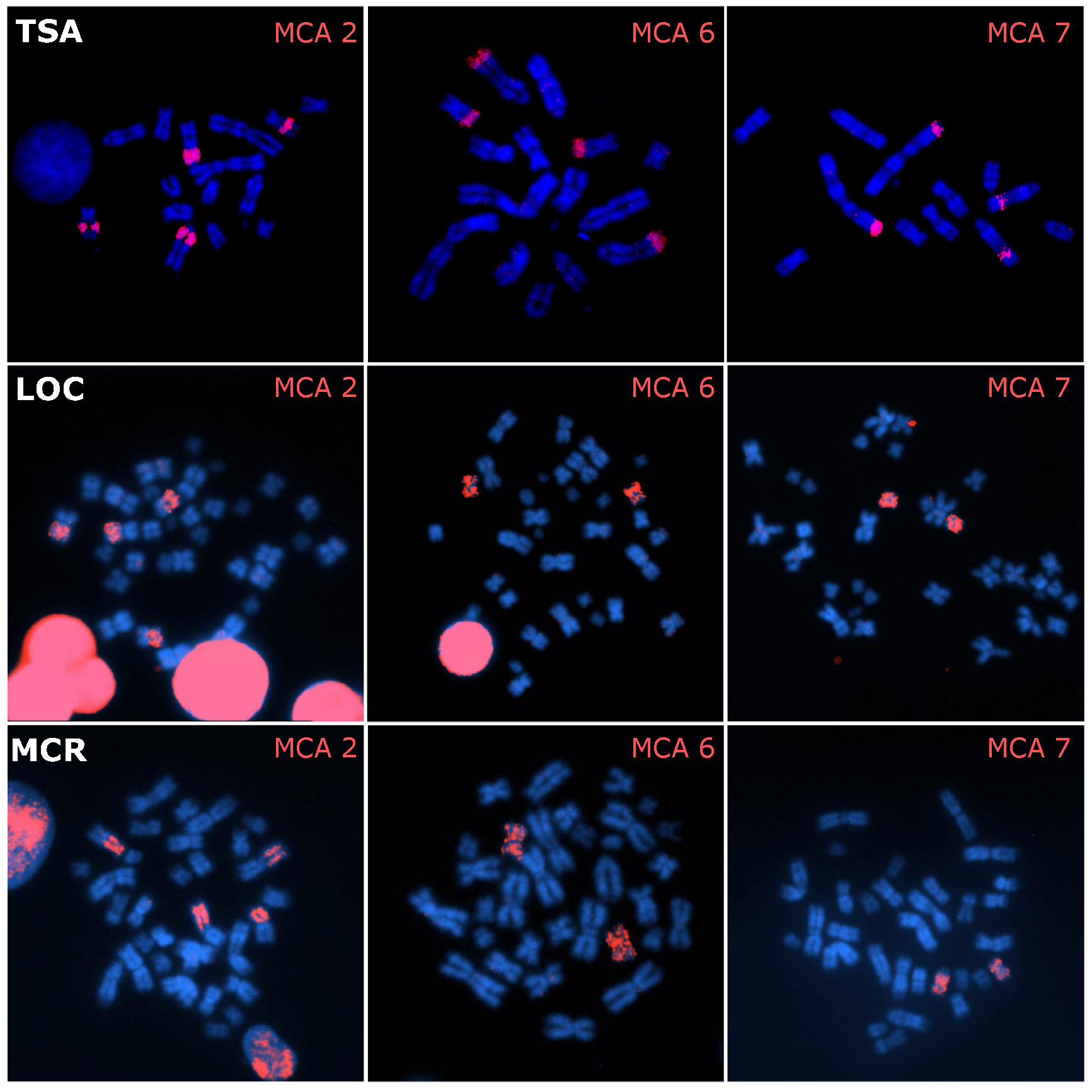

Supplement: Additional file 2: Figure S1. — Examples of in situ hybridizations using Macrotus californicus (MCA) chromosome paints on Phyllostominae metaphases. The MCA chromosome-specific probes used in TSA (a–c), LOC (d–f), and MCR (g–i) metaphases are indicated in pink in the upper-right corner of each picture. (JPEG 315 kb) [file 12862_2015_494_MOESM2_ESM.jpg]

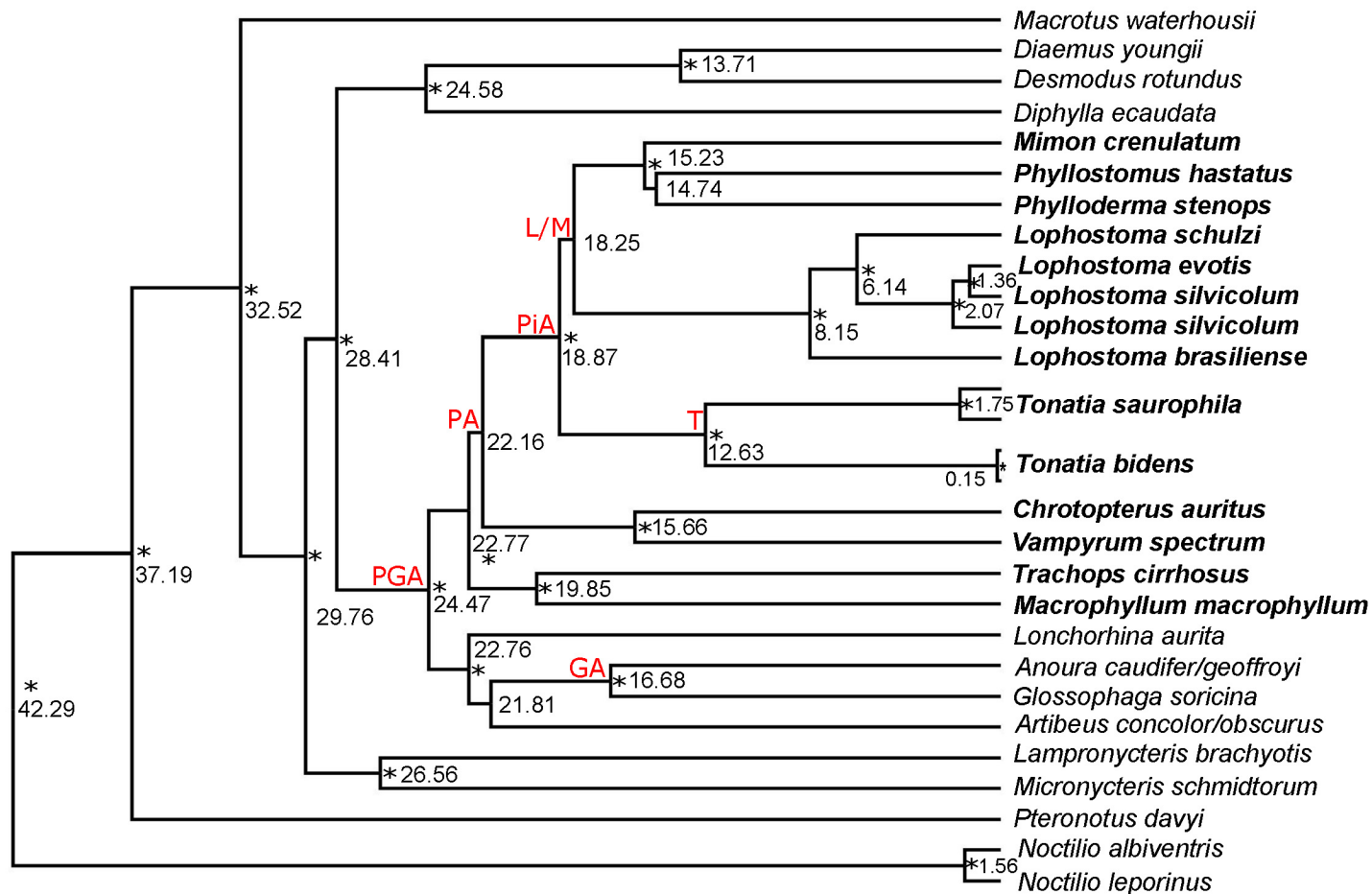

5.0

Supplement: Additional file 3: Figure S2. — Molecular phylogram depicting the relationships among genera of bats in the subfamily Phyllostominae (highlighted in bold) and the divergence time estimates (node ages) from BEAST analysis of all genes. Nodes with posterior probability values higher than 0.98 in (a) are marked with an *. Abbreviations for node names are as follows: Phyllostominae + Glossophaginae ancestor (PGA), Phyllostominae ancestor (PA), Glossophaginae ancestor (GA); Phyllostomini ancestor (PiA); Lophostoma + Mimon ancestor (L/M); genus Tonatia (T); genus Anoura (A). (PDF 1529 kb) [file 12862_2015_494_MOESM3_ESM.pdf]
