# Supplementary material for: Integration of molecular cytogenetics, dated molecular phylogeny, and model-based predictions to understand the extreme chromosome reorganization in the Neotropical genus Tonatia (Chiroptera: Phyllostomidae)
Source: BMC Evol Biol. 2015 Oct 6;15:220. doi: 10.1186/s12862-015-0494-y (PMC4594642; doi:10.1186/s12862-015-0494-y)
Supplement: Additional file 4: Tables S2–S5. — Detailed calculations of unique fusions and fissions for ACU and TSA and chromosomal associations shared between the analyzed species. (DOCX 29 kb) [file 12862_2015_494_MOESM4_ESM.docx]

**Additional file 4 – Tables S2-S5**

**Table S2: Syntenic blocks shared between six Phyllostomidae species.** The last column represents syntenic associations proposed to be present at the common ancestor (PGA) of the subfamilies Phyllostominae (LOC + MCR + TSA) and Glossophaginae (GSO + ACU).

| **MCA Syntenies** | ***Lophostoma occidentalis* (LOC)** | ***Mimon crenulatum* (MCR)** | ***Tonatia saurophila* (TSA)** | ***Glossophaga soricina* (GSO)** | ***Anoura cultrata* (ACU)** | ***Lonchophylla concava* (LCO)** | **PGA** |
| --- | --- | --- | --- | --- | --- | --- | --- |
| 10/5q | X | X | - | X | - | - | X |
| 12/2 | X | X | X | X | - | - | X |
| 13/3 | X | X | X | X | X | X | X |
| 14/1 | X | X | - | X | - | - | X |
| 3/11 | X | X | - | X | - | - | X |
| 2/1 | X | X | - | X | - | - | X |
| 4 | X | X | - | X | - | - | X |
| 5p | X | X | - | - | - | X | X |
| 6 | X | X | - | X | - | X | X |
| 7 | X | X | - | X | X | - | X |
| 8 | X | X | - | X | X | X | X |
| 9 | X | X | - | - | - | X | X |
| 15 | X | X | - | X | - | - | X |
| X | X | X | X | X | X | X | X |

**Table S3: Magnitude of unique fixed changes in the karyotypes of the Glossophaginae and Phyllostominae species analyzed.**

| **Taxon** | **Unique Fusions** | **Fissions from Subfamily Ancestral** | **Total** |
| --- | --- | --- | --- |
| *Glossophaga soricina* | 0 | 0 | 0 |
| *Anoura cultrata* | 8 | 6 | 14 |
| *Lophostoma occidentalis* | 0 | 0 | 0 |
| *Mimon crenulatum* | 0 | 1 | 1 |
| *Tonatia saurophila* | 26 | 13 | 39 |

**Table S4: List of fusion events required to form unique syntenic associations in TSA and ACU.**

| ***Tonatia saurophila*** | | | ***Anoura cultrata*** |
| --- | --- | --- | --- |
| 7/1 | 14/5 | 2/15 | 12/5 |
| 1/4 | 1/9 | 17/8 | 5/16 |
| 4/3 | 9/5 | 8/3 | 16/9 |
| 3/16 | 5/16 | 1/4b | 17/14 |
| 16/12 | 16/7 | 4/16 | 6/13 |
| 12/18 | 7/4 | 10/19 | 6p/1q |
| 18/13 | 4/6 | 19/8 | 19/18 |
| 2/10 | 8/11 | 8/10 | 18/ 15 |
| 10/14 | 11/2 |  |  |
| **Total: 26** | | | **Total: 8** |

**Table S5: Estimated number of syntenic group disruption in TSA and ACU.** The minimal number of chromosomal breaks required to derive the karyotype of *T. saurophila* and *A. cultrata* from the Phyllostominae and Glossophaginae ancestral karyotype was calculated based on the differential number of blocks a given MCA segment presented in ancestral subfamily, TSA and ACU karyotypes. (*) represents specific linkage groups with non-unique fission events shared by TSA and ACU.

| **MCA syntenic block** | **Number of blocks in Phyllostominae** | **Number of blocks in TSA** | **Fissions required TSA** | **Number of blocks in Glossophaginae** | **Number of blocks in ACU** | **Fissions required ACU** |
| --- | --- | --- | --- | --- | --- | --- |
| 1 | 2 | 3 | 1 | 2 | 2 | 0 |
| 2 | 2 | 2 | 0 | 2 | 2 | 0 |
| 3* | 2 | 3 | 1 | 2 | 2 | 0 |
| 4 | 1 | 3 | 2 | 1 | 2 | 1 |
| 5 | 2 | 2 | 0 | 2 | 2 | 0 |
| 6 | 1 | 2 | 1 | 1 | 2 | 1 |
| 7 | 1 | 2 | 1 | 1 | 1 | 0 |
| 8 | 1 | 3 | 2 | 1 | 1 | 0 |
| 9 | 1 | 1 | 0 | 1 | 2 | 1 |
| 10 | 1 | 3 | 2 or 1+inv | 1 | 1 | 0 |
| 11 | 1 | 1 | 0 | 1 | 1 | 0 |
| 12 | 1 | 2 | 1 | 1 | 1 | 0 |
| 13* | 1 | 2 | 1 | 1 | 2 | 1 |
| 14 | 1 | 1 | 0 | 1 | 1 | 0 |
| 15 | 1 | 1 | 0 | 1 | 1 | 0 |
| 16 | 1 | 2 | 1 | 1 | 2 | 1 |
| 17 | 1 | 1 | 0 | 1 | 1 | 0 |
| 18 | 1 | 1 | 0 | 1 | 2 | 1 |
| 19 | 1 | 1 | 0 | 1 | 1 | 0 |
| X | 1 | 1 | 0 | 1 | 1 | 0 |
|  | **Total TSA:** | | **13** | **Total ACU:** | | **6** |
